# Transcutaneous Electrical Nerve Stimulation Effects on Neglect: A Visual-Evoked Potential Study

**DOI:** 10.3389/fnhum.2013.00111

**Published:** 2013-08-19

**Authors:** Sabrina Pitzalis, Donatella Spinelli, Giuseppe Vallar, Francesco Di Russo

**Affiliations:** ^1^Department of Human Movement, Social and Health Sciences, University of Rome, Foro Italico, Italy; ^2^Neuropsychology Unit, IRCCS Santa Lucia Foundation, Rome, Italy; ^3^Department of Psychology, University of Milano-Bicocca, Milan, Italy; ^4^IRCCS Istituto Auxologico Italiano, Milan, Italy

**Keywords:** steady-state VEP, TENS, neglect, proprioceptive stimulation, neglect rehabilitation

## Abstract

We studied the effects of transcutaneous electrical nerve stimulation (TENS) in six right-brain-damaged patients with left unilateral spatial neglect (USN), using both standard clinical tests (reading, line, and letter cancelation, and line bisection), and electrophysiological measures (steady-state visual-evoked potentials, SSVEP). TENS was applied on left neck muscles for 15′, and measures were recorded before, immediately after, and 60′ after stimulation. Behavioral results showed that the stimulation temporarily improved the deficit in all patients. In cancelation tasks, omissions and performance asymmetries between the two hand-sides were reduced, as well as the rightward deviation in line bisection. Before TENS, SSVEP average latency to stimuli displayed in the left visual half-field [LVF (160 ms)] was remarkably longer than to stimuli shown in the right visual half-field [RVF (120 ms)]. Immediately after TENS, latency to LVF stimuli was 130 ms; 1 h after stimulation the effect of TENS faded, with latency returning to baseline. TENS similarly affected also the latency SSVEP of 12 healthy participants, and their line bisection performance, with effects smaller in size. The present study, first, replicates evidence concerning the positive behavioral effects of TENS on the manifestations of left USN in right-brain-damaged patients; second, it shows putatively related electrophysiological effects on the SSVEP latency. These behavioral and novel electrophysiological results are discussed in terms of specific directional effects of left somatosensory stimulation on egocentric coordinates, which in USN patients are displaced toward the side of the cerebral lesion. Showing that visual-evoked potentials latency is modulated by proprioceptive stimulation, we provide electrophysiological evidence to the effect that TENS may improve some manifestations of USN, with implications for its rehabilitation.

## Introduction

Transcutaneous electrical nerve stimulation (TENS) is a form of low-voltage stimulation historically used for therapeutic purposes, especially for pain relief (Sedan and Lazorthes, 1978; Dubinsky and Miyasaki, 2010; Rode et al., 2012). In the last decades, TENS was applied also in right-brain-damaged patients with left unilateral spatial neglect (USN), stimulating the contralesional side of the patient’s body, typically on the left neck muscles, but also on the left hand. Vallar et al. (1995) assessed the effects of TENS on left USN, using visual-motor exploratory tasks (letter cancelation): left neck stimulation temporarily improved the deficit in 13 out of 14 (93%) patients, while stimulation of the right neck had no positive effects, actually worsening exploratory performance in 9 (64%) patients. The temporary positive effects of left TENS extend to the left somatosensory deficits of right-brain-damaged patients, with and without left visual USN (Vallar et al., 1996). Right-sided TENS had no effects on the right somatosensory deficits of left-brain-damaged patients, with the exception of one left brain-damaged patient with right neglect, in whom the right somatosensory deficit was temporarily improved (Vallar et al., 1996). In sum, TENS may ameliorate both visual USN, and USN-related somatosensory deficits (Vallar, 1997, 1998). These beneficial effects of TENS on various manifestations of left USN have been confirmed by a number of successive studies (Guariglia et al., 1998, drawing by copy and from memory, shape comparison, familiar square description; Guariglia et al., 2000, spatial orientation by shape; Pérennou et al., 2001, neglect-related postural instability; see also Richard et al., 2001, for positive effects in patients with left USN on the rightward deviation of the straight ahead, with TENS delivered to the left sole; Beschin et al., 2012, with effects on both left USN and anosognosia for hemiplegia, although not in all tested patients). There is also evidence that TENS may be effective for rehabilitating left USN (Schröeder et al., 2008). One negative result is on record (Karnath, 1995). TENS, in sum, modulates, with direction-specific effects, a number of manifestations of the USN syndrome, as other side or direction-specific stimulations do (see reviews in Vallar, 1997; Rossetti and Rode, 2002; Kerkhoff, 2003; Rode et al., 2006; Chokron et al., 2007).

The specific mechanisms underlying these effects on a number of manifestations of the USN syndrome may include the restoration of defective representations of the side of space contralateral to the lesion (contralesional), and of the ability to orient spatial attention contralesionally, through complex patterns of activation of both the damaged right hemisphere (RH), and the contralateral left hemisphere, with differences related to the specific stimulation delivered to the patient (Bottini et al., 1995; Luauté et al., 2006; Saj et al., 2013). The directional-specificity of the effects of these stimulations on the different manifestations of the USN syndrome, as well as some evidence for their selectivity (Vallar et al., 1995, 1996), suggests that these effects cannot be considered “placebo” and that general cerebral activation is not the main mechanism supporting it.

In most of the studies showing amelioration of USN after TENS, the deficit was assessed in the visual modality, suggesting that the effects of the stimulation may extend to visual areas. There is electrophysiological (visual-evoked potentials, VEP) evidence from right-brain-damaged patients with left USN that the earliest responses of the RH striate and extra-striate areas to contralesional left-sided visual stimuli may be largely preserved (Vallar et al., 1991; Di Russo et al., 2008). Conversely, later right hemispheric electrophysiological activities in the visual areas (namely, parietal activity and top-down re-activation of extra-striate and striate areas) are reduced in amplitude, and delayed in latency, as compared with the corresponding activity in the left hemisphere (Di Russo et al., 2008, 2012). Such hemispheric differences decrease with recovery from USN following visual-spatial rehabilitation training (Di Russo et al., 2012). Thus, VEP hemispheric asymmetries appear a good marker of the reduction of USN. While Di Russo et al. (2012) focused on the effects of a diversified, multiple-inputs training procedure, lasting about 8 weeks (Pizzamiglio et al., 2006), the present study investigates the effects of a single, brief procedure of peripheral stimulation, namely TENS, at the level of visual cortical responses, to elucidate how the effects of TENS build up, as indexed by VEPs.

We used steady-state visual-evoked potentials (SSVEPs) because this technique is suitable under conditions of limited recording time (as in brain-damaged patients) allowing recording of 100 responses to stimulus repetition in about 1 min (conversely, transient VEPs would require 5–10 min). SSVEPs are the averaged responses to repetitive visual stimulation flickering at high temporal frequency; thus, they provide information about cortical activity patterns related to sustained visual experience. Indeed, the correlation between the SSVEP amplitude and psychophysical contrast threshold is a major indicator of the link between brain electrical activity and visual perception (Campbell and Maffei, 1970). A limitation of the SSVEP method is that, averaging together all different components of the visual response (which are, in contrast, well isolated by transient VEP) does not allow to discriminate between them. fMRI evidence shows that the major sources of SSVEP are V1 and MT/V5 (Di Russo et al., 2007). Nevertheless, as long as visual perception depends on the loop between early and higher-order visual areas, and on the combination of early activation and late re-activation of the same visual areas (e.g., Lamme, 2006), SSVEP, averaging all these activities, is a good candidate to represent an electrophysiological counterpart of visual perception.

Furthermore, previous studies in brain-damaged patients with left spatial neglect, based on SSVEP recording to stimuli located in the left and right visual half-fields (LVF and RVF), have shown that responses to LVF stimulation are delayed as compared with RVF stimulation (e.g., Spinelli et al., 1994). Finally, leftward rotation of the trunk – a maneuver than improves some manifestations of left USN – reduces the disproportionate longer latencies of SSVEP to visual stimuli delivered in the LVF of right-brain-damaged patients with left USN (Spinelli and Di Russo, 1996). In this study, we measured SSVEP asymmetries in right-brain-damaged patients with left USN before, immediately after, and 1 h after TENS.

## Materials and Methods

### Participants

Six right-brain-damaged patients with chronic left USN, and 12 healthy young controls (6 females, age 27.3 ± 2.3 years) participated in the study. Patients were recruited from the Neuropsychological Unit of the Santa Lucia Foundation, Roma, Italy. Demographic and clinical data of the patients are reported in Table 1. All patients had intact visual fields, based on standard kinetic perimetry. All patients had unilateral vascular lesions, summarized in Table 2. Lesions were large and heterogeneous, generally involving several cortical and sub-cortical areas. Patients with lesions involving the visual areas were not included. Only one patient had occipital damage (Table 2, Patient #2), which, however, did not involve early visual areas. As described in Table 2, areas V1 (BA17) and V2 (BA18) were totally spared, while extra-striate areas V3 and V3A (BA19) were mostly spared. Moreover, objective functional testing of visual responses to LVF stimuli showed in this patient the same electrophysiological pattern observed in the other patients. For these reasons the patient was included in the study. All participants were right-handed and had normal or corrected-to-normal visual acuity. Informed consent was obtained from each participant, and the study was approved by the local ethics committee of the Santa Lucia Foundation.

**Table 1:** **Demographic and clinical data for the neglect patients**.

Patient #	Sex/age	TFO	Line canc	Lett canc	WJ	Sent read	Line bisect
1	M/69	132	+	+	+	+	+
2	F/77	143	+	+	+	+	+
3	F/68	101	+	+	+	+	+
4	M/81	176	−	+	+	−	+
5	M/68	162	+	+	+	+	+
6	M/60	114	+	+	−	+	+
Mean	70.5	138					

*TFO, time from onset (days). Neglect tests: Line canc; line cancelation; Lett canc, letter cancelation; WJ, Wundt–Jastrow; Sent read, sentence reading; Line bisect, line bisection. The sign + identifies pathological performances according to standard normative values, while the sign − indicates performance above the cut-off (Pizzamiglio et al., 1989)*.

**Table 2:** **Lesion localization in the six neglect patients (see Materials and Methods for further details)**.

Patient #	Sites of lesions in the right hemisphere (RH)
1	Middle and posterior superior temporal gyrus, parahippocampal temporal gyrus, posterior half of cingulate gyrus
2	Inferior (supramarginal and angular gyri) and superior parietal lobule, superior temporal gyrus, mesial (supracalcarine) and lateral superior occipital region, occipital paraventricular area (areas 17 and 18 were totally spared, area 19 was mostly spared)
3	Superior temporal gyrus, precentral gyrus, and posterior sector of the frontal gyrus (primary and supplementary motor cortex), anterior cingulate cortex, pars opercularis of the frontal operculum
4	Precentral (primary sensory cortex), and frontal gyrus
5	Precentral and postcentral gyrus and posterior sector of frontal gyrus (primary sensory cortex, primary and supplementary motor cortex), superior temporal gyrus, posterior half of cingulate gyrus, inferior (supramarginal gyrus) parietal lobule, temporal pole, frontal operculum
6	Precentral and postcentral gyrus and posterior sector of the frontal gyrus (primary sensory cortex, primary and supplementary motor cortex), inferior (supramarginal gyrus) and superior parietal lobule, pars opercularis of the frontal operculum, superior temporal gyrus, posterior half of cingulate gyrus

### Behavioral tests

Patients performed the following tests:

1. Lines cancelation test (Albert, 1973). Participants were requested to cross 21 line segments randomly arranged on a sheet of white paper (11 on the left and 10 on the right). The score was the number of left and right crossed segments.
2. Letters cancelation test (Diller et al., 1980). Participants were requested to cross 104 letter H randomly arranged on a sheet of white paper (53 on the left- and 51 on the right-hand-side), intermingled with other distracter letters (a total of 208 non-targets). The score was the number of left- and right-sided crossed target letters.
3. Sentence reading test (Pizzamiglio et al., 1989). Six sentences of differing lengths were presented to each patient (e.g., The train goes from one city to another in 8 h) who was requested to read aloud each sentence. The score was the number of sentences correctly read. Hesitations, self-corrections or paralexias were not counted as errors.
4. Wundt–Jastrow area illusion test (Massironi et al., 1988). The stimuli were two semicircular fans of identical shape and size. Ten sizes (ranging from 6 to 58 cm), two orientations (upward-downward convexity), and two directions (leftward-rightward) were used, for a total of 40 stimuli. The participant’s task was to indicate which fan was longer. Responses were classified in two categories: “expected responses,” those consistent with the illusory effect in healthy participants; “unexpected responses” those not consistent with the illusory effect. The score was the number of “unexpected” responses, when the two fans were oriented toward the left or the right.
5. Line bisection (Albert, 1973). Participants were requested to mark with a soft pen the subjective midpoint of a 15 cm long and 1 mm wide horizontal line drawn on a centimeter paper. The test was repeated for 25 times. In each trial the participant’s deviation was measured to the nearest millimeter, scored as a leftward/rightward (−/+) deviation from the objective midpoint of the segment. The score was the average participant’s deviation from the objective midpoint.

The presence of USN was assessed using the first four tests, according to the standard neuropsychological battery of Pizzamiglio et al. (1989). Patients who failed on at least two out of four tests were classified as USN patients. For experimental purposes, four tests were administered pre- and post TENS stimulation (Line cancelation, Letter cancelation, Sentence reading, and Line bisection; see Data Analysis for further details). The Wundt–Jastrow Illusion was used for diagnostic purposes only.

### Electrophysiological measures

#### Stimuli

Stimuli were displayed on a monitor (Barco CDCT 6551) with mean luminance of 16.5 cd/m^2^ and frame rate 100 Hz. A cross in the center of the display served as fixation point. The stimulus was a horizontal sinusoidal 0.6 cpd grating of 80% contrast, 20° wide, and 20° high. The grating was displayed in separate runs in LVF and RVF. The edge of the grating was 1.5° to the fixation point. The steady-state VEP was elicited by grating contrast that was reversed sinusoidally at nine temporal frequencies (5, 5.5, 6, 6.5, 7, 7.5, 8, 8.5, 9 Hz).

#### VEP recordings

Visual-evoked potentials were recorded from scalp electrodes, Oz active with Cz as reference and Pz as ground. Signals were amplified (50,000-fold), band-pass filtered (1–100 Hz) and digitized at 64 points/period. The SSVEP waveform is roughly sinusoidal and is well described by the amplitude and phase of the second harmonic Fourier component. The SSVEP phase changes with temporal frequency; the apparent latency may be derived by measuring the phase as a function of temporal frequency, and estimating the slope of the curve (Spekreijse et al., 1977). The phase of the second harmonic is plotted in p radians as a function of temporal frequency under the assumption that phase advances or retard regularly with temporal frequency. Thus, multiple of 2p radians are added or subtracted to the raw data, in order to produce the maximum orderliness. The technique used in the present study was developed by Burr and Morrone (see Spinelli et al., 1994 for details). The computer performed on-line Fourier analysis to calculate the amplitude and the phase of the second harmonic component. At the same time, the computer averaged the electrical signals at a temporal frequency near that of the stimulus but not synchronously with it. This was taken as an index of noise and artifacts, to assess VEP reliability. For each packet of 20 sums (20 periods of stimulus presentation) the signal-to-noise ratio was calculated. As an independent measure of variability the standard error of the amplitude and phase was calculated from the two-dimensional scatter in amplitude and phase of the individual 20-sum packet. The apparent latency was estimated from the slope of the regression line of phases as a function of temporal frequency. The slope was calculated by least-squares fit, after weighting each data point by its signal-to-noise ratio.

### TENS application

Transcutaneous electrical nerve stimulation was applied to participants using an AGAR 2000™ stimulator with two disk electrodes (diameter 30 mm) located (15–20 cm apart) on the left superior trapezium muscle. The stimulation frequency was 100 Hz and the pulse duration was 100 μs. The mean intensity was 0.5 μA/mm. We did not include a right-sided TENS condition, since there is evidence that this side of stimulation is ineffective, or may actually worsen the deficit of USN patients, making the procedure unethical (Vallar et al., 1995, 1996).

### Procedure

The session started with the VEP recording to LVF and RVF stimuli, followed by the behavioral testing; four tests were administered to the patients, while healthy participants performed only the line bisection test (termed PRE condition). Then, the TENS was administered for 15 min. Immediately after TENS, VEPs to LVF stimuli were recorded, and the behavioral testing were administered again (POST condition). One hour after the termination of TENS, VEPs to LVF and the behavioral testing were administered again (POST60′ condition).

### Data analysis

#### Behavioral laterality score

For the line and letter cancelation tests, the laterality score was the difference between the number of canceled items on the left and on the right-hand-sides. Positive scores denoted more omissions in the left-half than in the right-half of the sheet. Reading errors were classified as left-sided or right-sided, depending to their position, with respect to the center of each sentence, which was aligned with the center of the sheet of paper. The laterality score was the difference between the number of errors in the left- and in the right-hand-side of the sentence. Positive scores indicated more reading errors in the left-half than in the right-hand-side of the sentence. Line bisection test directly expressed the value of asymmetry. Positive values indicated a rightward bias of the subjective midpoint. To verify the presence of asymmetry in the PRE condition, preliminary analyses compared the responses to left- and right-sided stimuli in behavioral tests. These scores in the PRE, POST, and POST60′ times were submitted to one-way ANOVAs.

#### Steady-state visual-evoked potential

It is known that, when comparing the LVF and RVF recordings of USN right-brain-damaged patients, the deficits are usually limited to LVF, while recordings to stimuli in the RVF are within normal limits (e.g., Di Russo et al., 2012). For this reason (as typically done in studies in brain-damaged USN patients) the more appropriate control of the LVF recordings are RVF recordings. Healthy participants were examined in this study just in order to assess the presence of the behavioral and electrophysiological effects of TENS in healthy people, not to compare their data with those of USN patients.

In order to assess the presence of VEP lateral asymmetries in the PRE condition, a preliminary analysis compared electrophysiological responses to LVF- and RVF- stimuli. Apparent latencies were submitted to one-way ANOVAs with Hemifield as factor. VEP amplitudes were submitted to a ANOVA with Hemifield and Temporal Frequency (nine levels 5–9 Hz) as factors. To evaluate the effect of TENS, the LVF amplitude at the peak, and the LVF apparent latency were submitted to one-way ANOVAs with the TENS factor at three levels (PRE, POST, and POST60′). An additional analysis used the values of asymmetry between LVF, tested in PRE, POST, and POST60′ conditions, and RVF baseline (PRE condition). Asymmetry was quantified for peak amplitude and apparent latency. The values of asymmetry were submitted to one-way ANOVAs with the TENS factor at three levels (PRE, POST, and POST60′).

In both behavioral and SSVEP analyses, *post hoc* comparisons were made using Newman–Keuls test. The overall alpha value was fixed at 0.05 after Greenhouse–Geisser correction.

## Results

### Behavioral data

**Figure 1:**
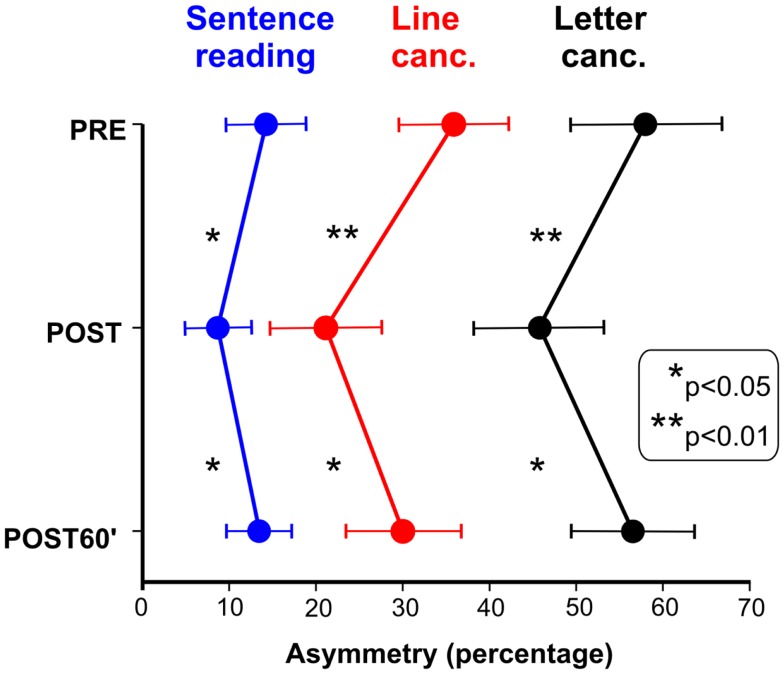
**Effect of TENS on neglect patients’ performances in sentence reading, line cancelation, and letter cancelation tests**. Scores: percent of omitted left minus right targets (positive values indicated more omissions in the left side of space).

**Figure 2:**
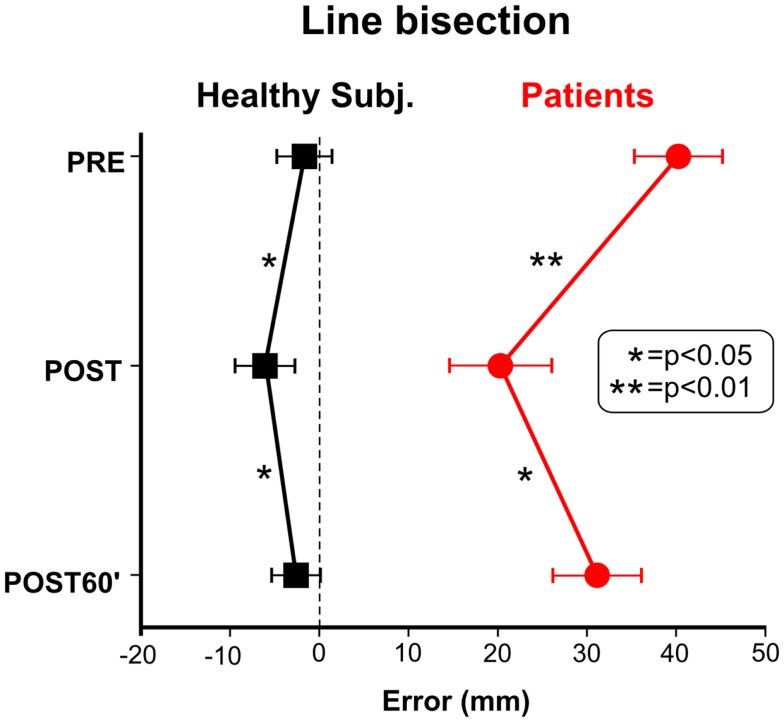
**Effect of TENS on line bisection performance of healthy subjects and neglect patients**. Positive values indicate a rightward shift of the subjective midline.

Figure 1 shows the effects of TENS in the reading and cancelation tests in the six brain-damaged USN patients; the right side of Figure 2 shows the patients’ average error (mm) in line bisection. In all tests we found a significant effect of TENS [*F*_(2, 10)_ > 5.53, *p* < 0.05] on performance asymmetry. *Post hoc* comparisons showed that the asymmetry was reduced after the TENS (PRE > POST, *p* < 0.05), and returned to the PRE stimulation level 1 h after it [POST < POST60′, *p* < 0.05]; PRE and POST60′ conditions did not significantly differ. Also in healthy participants the effect of TENS on the line bisection was significant [*F*_(2, 22)_ = 33.1, *p* < 0.0001]. The bisection error (left hand-side of Figure 2) was on average −1.5 mm before the stimulation; after TENS it was about −6 mm [only this latter value was different from the ideal performance (i.e., complete accuracy; *t*-test against zero, *t*_11_ = 3.3, *p* = 0.0034)]. One hour after TENS the mean deviation was about −2.5 mm. As for patients, the deviation in the POST condition differed from those in the PRE and in the POST60′ conditions (*p* = 0.0012), which did not differ from each other. In sum, the performance of the six right-brain-damaged USN patients improved in Line (44%), and Letter (19%) cancelation, in Sentence Reading (31%), and in Line Bisection (44%), after stimulation (post-treatment interval). On average, we found an improvement of 35% which is somehow comparable to the clinical amelioration found in previous studies using daily vibration TENS therapy (e.g., Johannsen et al., 2003: 25% in the Letter cancelation test and 29% in the Bell Test).

### Electrophysiological data

**Figure 3:**
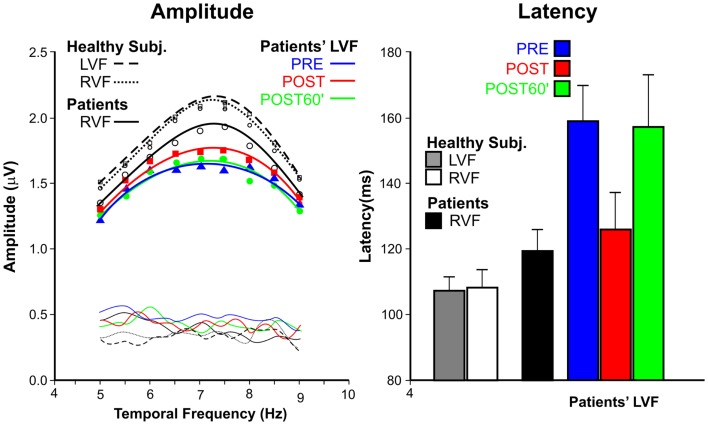
**Steady-state visual-evoked potential data**. Left panel: amplitudes as function of temporal frequencies for patients with spatial neglect and healthy subjects. For patients, the LVF responses are reported in PRE, POST, and POST60′ conditions. Thin lines without symbols represent the noise levels. Right panel: apparent latencies; as for amplitude, the data are reported in the three tested conditions.

Figure 3 show the average VEP amplitude (left panel) and apparent latency (right panel) superimposing the data of the patients’ and control groups, and, for patients, showing the data in the three conditions.

The amplitudes had the typical tuning function, with larger amplitudes around 7–8 Hz, and smaller amplitudes at lower and higher frequencies. The comparison between LVF and RVF amplitudes of the patients’ group in the PRE condition indicated that the difference was significant only at the peak of the functions (7.5 Hz), as shown by interaction between Hemifield and Temporal frequency *F*_(8, 40)_ = 3.9, *p* = 0.0018. For this reason only the peak amplitude was considered in the following analyses. The effect of TENS on the amplitude of the LVF responses of patients did not reach the significant level [*F*_(2, 10)_ = 2.16, *p* = 0.17]. Healthy participants, as expected, did not show any difference in the PRE condition between LVF and RVF; moreover, the effect of TENS was not significant (all *p*s > 0.54).

The apparent mean latency (right panel of Figure 3) in the PRE condition was 120 ms for the RVF and 160 ms for the LVF in USN patients; this difference was significant [*F*_(1, 5)_ = 22.45, *p* = 0.0051]. The effect of TENS on latencies was significant [*F*_(2, 10)_ = 52.9, *p* < 0.0001]; LVF response latencies in the POST condition (126 ms) were faster (*p*s < 0.0019) than in the PRE (160 ms) and POST60′ (157 ms) conditions. The latter two values did not differ from each other (*p* = 0.16). In healthy participants, LVF and RVF apparent latencies (both about 105 ms) did not differ from each other [*F*_(1, 11)_ > 1, ns]. The effect of TENS was significant [*F*_(2, 20)=_7.31, *p* = 0.0041]. The LVF response latency in the POST condition (98 ms) was shorter (*p* < 0.0063) than in the PRE (105 ms), and in the post POST60′ (104 ms) conditions, with the latter latencies being comparable.

**Figure 4:**
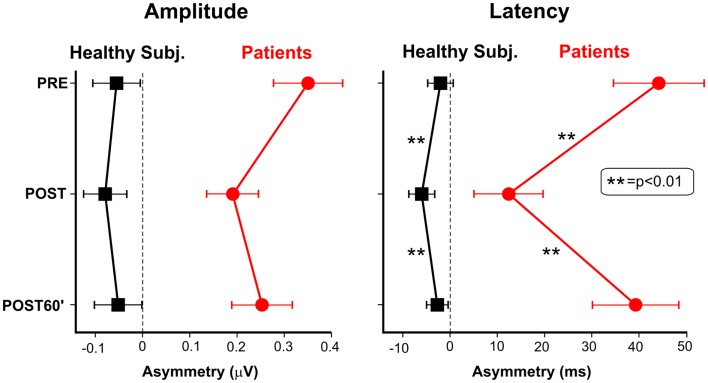
**Asymmetry of the cortical responses to stimuli in the two hemifields in healthy subjects and neglect patients**. Asymmetry is measured as difference between baseline RVF responses (PRE condition) and LVF responses measured in the three conditions (PRE, POST, and POST60′). The left panel shows the TENS effect on the amplitude at the peak temporal frequency (7.5 Hz). The right panel shows the TENS effect on apparent latency.

Figure 4 shows the VEP data as LVF-RVF asymmetries. Regarding the amplitude (left panel of Figure 4), in the patients’ group, TENS reduced the asymmetry, pushing the POST values toward the dashed vertical line (zero asymmetry). In healthy participants, the asymmetry tends to increase after TENS (the POST values shift away from the dashed vertical line), although the effect was not significant, both in patients and in healthy participants (all *p*s > 0.49). Regarding the apparent latency, TENS significantly modulated the asymmetry in USN patients [*F*_(2, 10)_ > 19.27, *p* = 0.0004]. The asymmetries of both the PRE and the POST60′ conditions were larger than that of the POST condition (*p* < 0.0005), which did not differ from each other. Also in healthy participants, TENS modulated the hemifield asymmetries [*F*_(2, 22)_ = 17.7, *p* = 0.0003]. The asymmetry in the POST condition (6 ms) was larger (*p* < 0.015) than the other two conditions, which did not differ each other. In summary, patients showed an average improvement of 22% in the VEP latency asymmetry, after stimulation (post-treatment interval).

## Discussion

The present results first confirm previous observations (Vallar et al., 1995, 1996; Guariglia et al., 2000), showing that TENS brings about a temporary amelioration of left USN, as measured by standard clinical tests. Notably, the present findings are unlikely to reflect a sort of placebo effect. Contrary to this interpretation, there is evidence that the effects of TENS crucially depend on the side of the input, namely: left, but not right, neck stimulation is effective in temporarily reducing both left USN as assessed by visuo-spatial exploratory tasks (Vallar et al., 1995), and the USN-related component of somatosensory deficits (Vallar et al., 1996).

Second, we report a novel finding, namely an effect of TENS on the electrophysiological cortical activity evoked by stimuli in the left “neglected” half-field. Indeed, in the PRE condition, the apparent latency of VEPs to LVF stimuli was longer than to RVF. After TENS the LVF latency became much shorter (with an average reduction of 22%). A similar, although not significant, trend was present also for signal amplitude, which appears to be a less sensitive index in this respect (see discussion in Di Russo and Spinelli, 2002). Indeed, most of the studies investigating SSVEPs in right-brain-damaged patients with left USN found increased latencies for LVF stimulation, with no effects (Spinelli et al., 1994) or less specific effect (Angelelli et al., 1996) on amplitude.

One may wonder whether such an electrophysiological result reflects a TENS modulation of early or late visual processing. There is evidence from two electrophysiological studies with transient VEPs, using large electrodes array and focal stimuli in the four visual quadrants, which allow a fine discrimination of the VEP components (Di Russo et al., 2008, 2012), that the early components (peaking at 75 and 100 ms) are largely preserved in non-hemianopic USN right-brain-damaged patients. This suggests that visual processing in early striate and extra-striate areas is preserved. In contrast, the visual components peaking at 130, 180, and 250 ms show a definite left-right asymmetry. Furthermore, there is evidence (Di Russo et al., 2012) that early components are not affected by visual-spatial training, which, in turn, reduces the hemispheric asymmetry of the later components. SSVEPs do not allow to isolate different processing levels; indeed, by averaging responses across time, and overlapping bottom-up and top-down activities, they provide a single, overall, value of latency related to the neural processing that takes place in the visual areas (e.g., Störmer et al., 2013). So, at which level the reduction of the lateral spatial asymmetry characterizing USN may occur? The present experiment cannot exclude a direct effect of TENS on the early responses of the visual cortices; however, taking into account the values of response latency to stimuli displayed in the LVF before (160 ms) and after (126 ms) TENS, it seems likely that an important portion of the effect is due to post-sensory components. The bottom-up 130 component (possibly generated in dorsal IPS, and representing a likely candidate in the hemispheric race for priority, Marzi et al., 2000), and the top-down re-entrant feedback on striate and extra-striate areas (components peaking at 180 and 250 ms) might contribute to the effect.

The suggestion has been made (Corbetta et al., 2005) that the dorsal parietal system, anatomically intact in most USN right-brain-damaged patients (Vallar, 2001; Committeri et al., 2007), is dysfunctional as a consequence of damage to the ventral posterior parietal regions (i.e., the inferior parietal lobule). In the present study we observed behavioral and electrophysiological asymmetries in the horizontal meridian space. This was shown in patients without hemianopia and without lesions in early visual areas (see Table 2). Therefore, the USN patients’ performance cannot be attributed to the inability to compensate for a visual field deficit occurring at an early stage, such as in patients with left USN and left hemianopia (e.g., Doricchi et al., 2003). There is evidence from both monkeys (e.g., Galletti et al., 1996; Page and Duffy, 2003) and humans (e.g., Sereno and Huang, 2006; Bolognini and Maravita, 2007; Smith et al., 2012; Pitzalis et al., 2013), that a number of dorsal parietal areas (VIP, V6A, 2v) are involved in integrating vestibular, somatosensory, and visual inputs. This multimodal dorsal parietal network may receive additional strong and asymmetric inputs by TENS, and would temporarily enhance feedback activity to right-sided visual areas, increasing the saliency of LVF stimuli, and partially and temporarily reducing the pathological unbalance toward the right side. This dorsal network of multimodal parietal areas may constitute a basis for the building up and updating of non-retinal representations of space (e.g., Johannsen et al., 2003). TENS to the left posterior neck muscles can be regarded as a bottom-up activation of these higher-order transformation processes. As shown in Table 2, the superior parietal lobule was structurally damaged in two out of six patients (#2 and #2), and largely spared in the remaining four patients. In conclusion, dorsal posterior parietal (typically structurally spared in USN patients) regions may support the effect of TENS measured with SSVEPs; future studies, using high-resolution multi-channels VEP recordings, may assess these hypotheses.

After TENS, healthy participants make a leftward error (TENS effect about 5% of the line length). Thus, they show similar effects, although much minor in size, than those exhibited by USN patients (about 1.4%). In addition, we found that TENS was associated to a reduction of the LVF VEP latency, which was 6 ms earlier than RVF.

It may be noted that, before applying TENS, healthy participants show a leftward (although not significant) deviation in line bisection. This phenomenon has been repeatedly found both when participants see the line, and when they are blind-folded, relying only on tactile and kinesthetic information (“pseudoneglect,” see Jewell and McCourt, 2000). The phenomena of neglect and pseudoneglect are considered manifestations of a common underlying attentional asymmetry (Pitzalis et al., 2001). The present data show that both phenomena are affected by TENS, thus supporting view (see Discussion in Jewell and McCourt, 2000) that they share some basic mechanisms.

A final remark concerns the implications for the neuropsychological rehabilitation of USN patients. The different techniques proposed through the years to rehabilitate neglect can be distinguished in two main categories of approaches: top-down and bottom-up. Top-down techniques attempt at actively re-orienting the patients’ attention toward the neglected left side of space. Bottom-up techniques, conversely, consist in delivering asymmetrical sensory stimulations, which do not require the patients’ active participation in exploring the neglected side of space (see Vallar and Bolognini, 2011; Zoccolotti et al., 2011 for review). TENS, which is a bottom-up technique, may bring about a passive activation of the neglected side of the body, thus potentially compensating for the rightward bias of neglect (e.g., Vallar et al., 1995, 1996; Guariglia et al., 2000). With respect to top-down techniques (which require patients to be aware of their deficits, and to be able to voluntarily maintain attention oriented toward the affected side), treatments based on bottom-up mechanisms are potentially more successful because they are tied to less prerequisites concerning the functional status of USN patients (i.e., they do not necessarily require the patient’s cooperation in attending and exploring the left hand-side of space). Furthermore, TENS or neck muscle vibration have the advantage of being suited for stimulus application anywhere and anytime, even at home after discharge from the hospital. Also, these techniques have no side-effects and are easy to apply. It thus seems to be a useful tool to supplement the established methods in the rehabilitation of spatial neglect.

In conclusion, VEP apparent latency and behavioral performance in patients with neglect can be modulated by TENS stimulation which is able to induce a deficit reduction of valuable magnitude; the observed effects regress 1 h after treatment. Also healthy subjects are sensitive to TENS, showing effects similar to patients group, but much less intense. The present study confirms that TENS is a technique potentially useful in the field of neuropsychological rehabilitation.

## Conflict of Interest Statement

The authors declare that the research was conducted in the absence of any commercial or financial relationships that could be construed as a potential conflict of interest.
